# Impact of Covid-19 Lockdown on Availability of Drinking Water in the Arsenic-Affected Ganges River Basin

**DOI:** 10.3390/ijerph18062832

**Published:** 2021-03-10

**Authors:** Srimanti Duttagupta, Soumendra N. Bhanja, Avishek Dutta, Soumyajit Sarkar, Madhumita Chakraborty, Ashok Ghosh, Debapriya Mondal, Abhijit Mukherjee

**Affiliations:** 1 Graduate School of Public Health, San Diego State University, San Diego, CA 92182, USA; srimanti.duttagupta@gmail.com; 2 Interdisciplinary Centre for Water Research, Indian Institute of Science Bangalore, Karnataka 560012, India; soumendrabhanja@gmail.com; 3 Scripps Institution of Oceanography, University of California, San Diego, La Jolla, CA 92037, USA; avishekdutta14@gmail.com; 4 School of Environmental Science and Engineering, Indian Institute of Technology Kharagpur, West Bengal 721302, India; soumyajit.sarkar3@gmail.com; 5 Department of Geology and Geophysics, Indian Institute of Technology Kharagpur, West Bengal 721302, India; madhumitageol@gmail.com; 6 Bihar State Pollution Control Board, Patliputra, Patna, Bihar 800010, India; ashok.ghosh51@gmail.com; 7 Mahavir Cancer Institute and Research Centre, Phulwari Sharif, Patna, Bihar 801505, India; 8 Centre for Clinical Education, Institute of Medical and Biomedical Education St George’s, University of London, London SW17 0RE, UK; dmondal@sgul.ac.uk

**Keywords:** Ganges river, COVID-19, water quality, lockdown, BOD, COD, fecal coliform

## Abstract

The 2020 COVID-19 pandemic has not only resulted in immense loss of human life, but it also rampaged across the global economy and socio-cultural structure. Worldwide, countries imposed stringent mass quarantine and lockdowns to curb the transmission of the pathogen. While the efficacy of such lockdown is debatable, several reports suggest that the reduced human activities provided an inadvertent benefit by briefly improving air and water quality. India observed a 68-days long, nation-wide, stringent lockdown between 24 March and 31 May 2020. Here, we delineate the impact of the lockdown on groundwater and river sourced drinking water sustainability in the arsenic polluted Ganges river basin of India, which is regarded as one of the largest and most polluted river basins in the world. Using groundwater arsenic measurements from drinking water wells and water quality data from river monitoring stations, we have studied ~700 km stretches of the middle and lower reaches of the As (arsenic)-polluted parts of the river for pre-lockdown (January–March 2020), syn-lockdown (April–May), and post-lockdown periods (June–July). We provide the extent of As pollution-free groundwater vis-à-vis river water and examine alleviation from lockdown as an opportunity for sustainable drinking water sources. The overall decrease of biochemical oxygen demand (BOD) and chemical oxygen demand (COD) concentrations and increase of pH suggests a general improvement in Ganges water quality during the lockdown in contrast to pre-and-post lockdown periods, potentially caused by reduced effluent. We also demonstrate that land use (agricultural/industrial) and land cover (urban-periurban/rural) in the vicinity of the river reaches seems to have a strong influence on river pollutants. The observations provide a cautious optimistic scenario for potentially developing sustainable drinking water sources in the arsenic-affected Ganges river basin in the future by using these observations as the basis of proper scientifically prudent, spatially adaptive strategies, and technological interventions.

## 1. Introduction

The year 2020 started with a world-wide pandemic of immeasurable scale. The surfacing of the infection caused by the unexpected and unprecedented virus, COVID-19, has altered life to a ‘new normal’ [1]. As the virus started spreading, it reached every continent, possibly except the Antarctic, and the extent of the infection, its coverage and the number of fatalities reported was ~30.7 million infected persons, with ~1 million fatalities up to September 20, 2020 in 183 countries and 33 territories [2]. The pandemic is not only a global public health emergency but also has huge socio-economic-environmental ramifications, with potential long-term effects on the pathways to attaining sustainable development goals like access to clean drinking water.

India, the second-most populous country of the world, also is facing the brutal brunt of the pandemic and presently ranks among one of the highest countries in terms of the number of infected people (>5 million) and more than 84,000 deaths [2]. The Indian government initiated an unprecedented, preemptive 68-day long, nation-wide lockdown, starting from March 24 and ending on 31 May 2020 [3,4]. The complete nation-wide lockdown led to an absolute restriction of human activities in terms of public life, transportation, business, and industrial activities. While numerous studies on COVID19 have linked air quality improvement in parts of India [5], very few studies report water quality issues at sporadic locations [6,7].

Access to clean drinking water and sanitation is regarded as one of the primary goals of sustainable development across the globe. With its vast population and low per-capita income, a large portion of Indians still lack access to clean and sustainable drinking water [8,9]. Uncontrolled discharge of industrial waste and domestic sewage has rendered major rivers like the Ganges extremely polluted, and groundwater is rapidly depleting due to pervasive abstraction. Further, groundwater in several parts of the country contains elevated, toxic concentrations of geogenic, naturally occurring, non-point sourced pollutants, e.g., arsenic (As), which according to recent estimates has exposed at least 100 million residents of these groundwater polluted areas to suffer from As-related health hazards [10,11,12,13]. The extent and severity of groundwater As contamination of the Ganges river basin covering major parts of the states of Uttar Pradesh, Bihar and West Bengal has been ascribed as the largest mass poisoning in human history.

Thus, the Ganges river basin, both river and groundwater, has been under extreme stress for several decades. Discharge of untreated, poisonous effluents into the Ganges river from numerous industries (chemicals, fertilizer, food processing, jute, heavy industries, automobiles, etc.), and the functional failure of water treatment plants in hundreds of urban centers (Kanpur, Varanasi, Allahabad, Patna, Bhagalpur, Berhampur, Krishna Nagar, Kolkata, Howrah, etc.), non-point source discharge from irrigational practices (paddy, jute, sugar cane, pulses, corn, jute, etc.), the river navigation setup, power plants, etc., has been distressing for hundreds of millions of lives on its alluvial plains [14]. Further, the unconsolidated sediments underlying the Ganges River plains form a prolific aquifer system which holds one of the largest groundwater reserves in the world and includes a major portion of the freshwater demands for drinking water for millions of people inhabiting these plains. In 1986, the Government of India initiated the Ganga Action Plan (GAP) with a specific objective of controlling point-sourced industrial pollution, non-point-sourced pollution from agricultural run-off, cattle and human defecation, and disposal of carcasses and other organic wastes, etc. [14]. However, even after immense effort in alleviating river water quality, there were barely any long-term tangible positive outcomes, but rather water quality deteriorated. Subsequently, in 2014, an advanced version of GAP was launched as Namami Gange Mission (NGM) with the aim of integrated river conservation [14]. However, so far, the desired outcome has not been documented. In both these programs and other allied initiatives, one of the primary goals was to remediate Ganga river water quality to potable standards.

The shift from surface water to groundwater as the primary source of drinking and irrigation water has surely decreased water-borne enteric disease cases but has also resulted in prolonged exposure of millions of people to groundwater arsenic contamination, causing severe mass-poisoning and long-term public health concerns.

However, the lockdown provided a unique and near-hypothetical opportunity to delineate the difference in water quality observed in a scenario with limited or no industrial and human activity, potentially close to that of the pre-industrial period. However, detailed scientific study using geospatial and statistical analyses of the various datasets for sustainable drinking water availability in the groundwater arsenic-polluted part of the Ganges river basin, establishing causal relationships through human interferences, is still largely unavailable.

Hence, the present work develops an understanding of the available drinking water resources in the alluvial basin where the river water is known to be terribly polluted by human-sourced pollutants and groundwater is polluted by geogenic arsenic. The goals of the study are thus to (1) delineate the spatial extent and intricacies of the arsenic pollution in groundwater and the extent of contaminated river water reaches in the non-pandemic period, thereby identifying potable water sources in the region; (2) understanding the impact of cessation of human activity due to pandemic-related lock-down on river water quality, thereby modifying its suitability as a drinking water source; and (3) examining the experience gathered due to the un-precedented lockdown in determining drinking water sources in these known groundwater arsenic-polluted areas.

Using publicly available data, we report, evaluate, and discuss drinking water sustainability in the catchment of the Ganges river, using groundwater As measurements of drinking water wells and water quality changes at 19 locations in the middle and lower reaches of the river channel, spaced across a ~700 km long stretch, for the lockdown period in comparison with preceding and succeeding time periods. The specific objectives of this study are to identify, delineate and quantify the impact of the lockdown period on drinking water sustainability and availability during pre-lockdown (January–March 2020), lockdown (end of March–May 2020) and post-lockdown periods (June–July 2020) in the arsenic affected Ganges river basin.

## 2. Methods

### 2.1. Study Area

**Figure 1:**
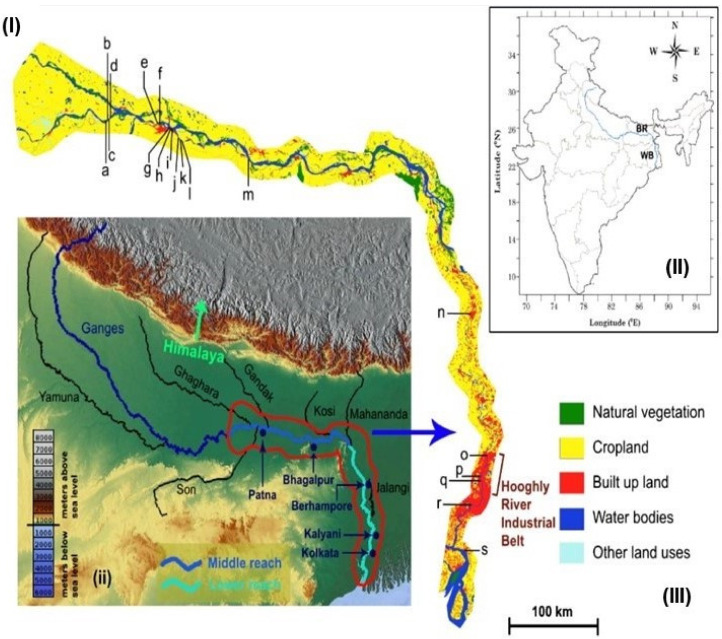
Map of the study area, displaying (**I**) Ganges river basin in India and the two Indian states within the study area, Bihar (BR) and West Bengal (WB); map of the Ganges river (blue line) and its major tributaries (black line) (**ii**); in foreground, the topography of parts of northern and eastern India (**II**). The study sites in the middle and lower reaches of the Ganges are marked in the map. Major cities/towns within the study are also marked; (**III**) land use and land cover map are shown (data for 2005 at 100 m resolution are collected from the Oak Ridge National Laboratory archive [16]. The water quality monitoring locations along the Ganges river are marked as: a: Chausa Buxar; b: Jail Ghat Buxar; c: Ramrekha Ghat Buxar; d: Buxar Bridge; e: Danapur Pipapul; f: Kurji Dargah; g: NIT Patna; h: Gulbi Ghat; i: Gai Ghat; j: Kachchi Dargah; k: Triveni Ghat Fathua; l: Kewala Ghat; m: Begusarai Simaria Ghat; n: Berhampore; o: Kalyani; p: Palta; q: Sreerampore; r: Garden Reach; s: Diamond Harbour.

The Ganges river basin, the sixth largest fluvial system in the world, accounts for about one-fourth of India’s total geographical area and is the biggest river basin in India, covering the entire states of Uttarakhand, Uttar Pradesh (UP), Bihar, Delhi, and parts of Punjab, Haryana, Himachal Pradesh, Rajasthan, Madhya Pradesh, and West Bengal [15]. Most of the middle reach and lower reach, which are the study areas for this work (Figure 1), pass through the states of Bihar (25.74°N/88.68°E to 25.30°N/87.62°E) and West Bengal (25.19°N/87.80°E to 21.98°N/88.10°E), before it discharges into the Bay of Bengal. The major tributaries of the Ganges in Bihar include the Son, Gandak, Ghaghara and Kosi rivers, and in West Bengal the Jalangi and Mahananda rivers (Figure 1). In addition to the water brought in by the tributaries, most of the river flow of the middle and lower reaches is sustained by groundwater baseflow and monsoon precipitation [14].

The plains of the Ganges river are regarded as one of the most fertile terrains of South Asia, and hold a huge reserve of groundwater, thereby being intensely used for crop irrigation. More than 450 million people inhabit the Ganges river basin, of which about 100 million live in several of the big cities located on the riverbank, while 350 million live mostly in agriculture-dominated rural areas. Bihar and West Bengal have a population density of more than 1000 people/km^2^, contributing to more than 200 million of this population [15]. The major parts of the Ganges riverine plain in Bihar are rural to peri-urban, with few large towns, e.g., Bhojpur, Patna and Bhagalpur. The West Bengal part of the river basin is much more urbanized (Figure 1), with several large towns like Malda, Behrampur, Krishnagar, Kalyani, Naihati, and the mega-city of Kolkata. Several industrial zones are also located along the Ganges river in Bihar and West Bengal, including the well-known, colonial era Hooghly industrial belt in West Bengal, which was also known as the “Manchester of the East”. The industries include thermal plants, chemical industries, distilleries, food, dairy and beverages, paper and pulp, sugar, jute, rubber, textile, bleaching and dyeing, tanneries, etc. Most have a waste outlet to the river, thereby severely polluting the river water.

### 2.2. Data Acquisition and Analyses

#### 2.2.1. Ganges River Water Quality

In order to assess the long-term trends in changing Ganges river water quality, monthly data were acquired from 19 locations from the middle and lower reaches of the Ganges river. Among them, 13 locations in the middle reach and six locations in the lower reach of the Ganges river were considered for this study (Figure 1, Table S1). Continuous data were considered for further analysis. Five parameters, namely biochemical oxygen demand (BOD, mg/L), chemical oxygen demand (COD, mg/L), fecal coliform concentration (FC, MPN/100mL), pH, and total dissolved solids (TDS, mg/L) were continuously monitored on a monthly basis from March to July 2020 in thirteen locations of the middle reach of the Ganges river (Table 1 and Table 2). Water quality parameters were estimated by following American Pharmacists Association (APHA) Standard Methods (23rd edition, 2017) [17]. These long-term spatial data of the middle and lower reaches of Ganges river water were obtained from Bihar Pollution Control Board and West Bengal Pollution Control Board websites, respectively [15,18]. To make the data statistically robust, the third quartile was calculated for each block for all the years for a particular parameter [8]. To omit outliers in the data, Tukey’s fencing method was used [8,19]. The outlier limit was calculated by multiplying the third quartile value by 1.51. Data exceeding the outlier limit were ignored whereas values lesser than this limit were kept unaltered. The resulting set of cleaned data for the study period and area was used for the various analyses.

#### 2.2.2. Groundwater Arsenic and Water Quality

In order to demarcate the extent of elevated As concentrations (exceeding the WHO—World Health Organization permissible limit of 10 µg/L) in groundwater and identify the areas with groundwater arsenic concentrations below 10 µg/L in the study area, we have analyzed the spatial patterns of groundwater As occurrence and developed a machine learning model to predict the distribution of As in groundwater. For this, we compiled a total of 518,639 field observations of groundwater As from Bihar and West Bengal. Annual data were collected from the database of the National Rural Drinking Water Program (NRDWP), Ministry of Jalshakti (erstwhile Ministry of Drinking Water and Sanitation and Ministry of Water Resources, River Development, and Ganga Rejuvenation), Government of India (*n* = 112,308 and *n* = 405,910 drinking water wells from Bihar and West Bengal, respectively) (NRDWP, 2018) and from the Central Ground Water Board (CGWB), Ministry of Jal Shakti, Government of India (*n* =112 and *n* = 309 monitoring wells from Bihar and West Bengal, respectively) (CGWB, 2018) [20]. The NRDWP monitors drinking water quality by sampling waters from its network of nearly three million wells, spreading across 32 states and union territories of the country and, in general, sampling is carried out from these wells once to multiple times per year. An initial pre-processing of collected data was performed to make the data clean for continuity and robustness by removing outliers from the dataset. This pre-processing resulted in a final dataset of 428,951 groundwater As data, which was used for the delineation of As occurrence and development of a model to predict its distribution.

### 2.3. Assessment and Predictions

#### Risk Analysis and Random Forest Modeling

To develop the groundwater As prediction model, the final dataset of 428,951 groundwater As data was discretized into grids with a resolution of 1000 m (x) × 1000 m (y) for the two states. The geometric of mean As data falling within the same grid was calculated to achieve uniform distribution of As over the study area. This discretization resulted in a final 53,346 (22,432 and 30,914 from Bihar and West Bengal, respectively) groundwater As data points, which were used for development of the prediction model. Binary coding of the data was performed based on the WHO permissible value of 10 µg/L for As in drinking water, i.e., 0 for <10 µg/L (or low risk) and 1 for ≥10 µg/L (or high-risk). About 94% of the data in this final dataset was 0 or low-risk and about 6% was 1 or high-risk.

For the development of the prediction model, a Random Forest (RF) model was used. RF is a non-linear approach to constructing a large number of de-correlated and random decision trees, where the final output is the ensemble of the outputs of all the trees in the forest, and therefore known as an ensemble learning technique [21]. The final dataset of 53,346 was spilt on an 80:20 ratio for training and testing of the model. A 10-fold cross-validation was used to maximize the model accuracy [22,23]. A set of 26 predictor variables for climate, geologic-geotectonic, hydrology, soil, and anthropogenic categories was initially selected based on the literature and the presumed influence on groundwater As [24]. These 26 variables were tested for correlation with groundwater As using Spearman’s rank correlation test and univariate logistic regression. Out of 26 variables, 14 satisfied both tests at 95% confidence interval and were considered for the final RF model development [24]. The final RF model was built using 1000 trees, the number of variables at each node to be picked randomly was fixed as 3 (square root of the number of predictor variables), and one-third of the training data was picked randomly, and used as out-of-bag (OOB) data for calculating model accuracy. Due to the imbalanced nature of the dataset, down-sampling with replacement was used to achieve a balanced model performance. The RF model also calculated the relative variable importance of the variables based on the mean decrease in model classification accuracy and Gini node purity [24]. Random forest modeling and associated statistical tests with modeling were executed in the R programming environment (version 3.6.1, R Foundation, Vienna Austria). The RF model was performed using “randomForest” package [22,23], while Spearman’s correlation test and univariate logistic regression test were performed using “stats” and “glm2” (Glm2, 2018) packages, respectively [22].

We have estimated the population with no risk of exposure to elevated As concentrations and also the population exposed to high As concentration (≥10µg/L) in groundwater, assuming groundwater as the primary resource of drinking water for the people residing in the study area, besides the possibility of irrigational use. Therefore, elevated As indicates directly a toxic As hazard. The estimations of population exposure were carried out using the projected populations for the year 2015 based on the spatial distribution of groundwater As reflected in the field-observed data and also for the model-predicted high groundwater As areas [25,26].

### 2.4. Statistical Analysis

#### 2.4.1. Correlation and Multivariate Analyses for Pre-, During- and Post-Lockdown Periods

Data organization, Pearson correlation, and heatmap construction were carried out in R and R Studio [27]. Pearson correlation was used to identify the difference between pre-lockdown (March 2020), during lockdown (April–May 2020), and post-lockdown (June–July 2020) periods for five parameters (Fecal coliform, BOD, COD, pH, and TDS) from the middle reach of the Ganges (Bihar, India). Similar analyses were conducted to find the difference between pre-lockdown (January–March 2020), during lockdown (April–May 2020), and post-lockdown (June 2020) period for eight parameters (Fecal coliform, BOD, COD, pH, sulfate, phosphate, nitrate, and TDS) from the lower reach of the Ganges (West Bengal, India). Heatmap and cluster analyses based on monthly average data of different aforementioned parameters across the middle and lower reaches of the Ganges were carried out with the pheatmap package [27]. Clustering of different phases was done based on a correlation matrix using the complete-link method.

Non-metric multidimensional scaling (NMDS) of the Euclidean distance of the four measured parameters (pH, BOD, COD, and fecal coliform) during lockdown across all the locations from lower and middle reaches of the Ganges was done using the phyloseq package in R [28]. This analysis was carried out to compare the effect of land use on river pollution in the lower and middle reaches of the Ganges.

#### 2.4.2. Panel Data Analysis

This study involves panel data analysis to account for individual heterogeneity using STATA v. 4.2 (StataCorp LLC, Texas, USA). Panel data refer to sets that consist of both time and series and cross-sectional data [9,29]. In this study, the fixed effect panel data was considered. It is assumed that the error component and the X’s are uncorrelated [30]. Fecal coliform concentration was considered as a dependent variable, and BOD, COD, pH, and sulfate were considered as independent variables. The comparison between the dependent and the independent variable is done by means of t-statistics. Some other assumptions and limitations are provided below: Strict exogeneity: E (∈it|Xi, αi) = 0, E(∈it|Xi, αi) = 0 (i.e., the idiosyncratic errors are uncorrelated with the covariates and the fixed effects).

### 2.5. Assumptions and Limitations

The data are obtained from the aforesaid mentioned sources and are presented based on their availability. They are based on observation of the discrete sampling points, and their representative interpolation across the entire studied reaches of the Ganges river is based on scale-dependent observations. Discussion of the fate of solutes and other water quality parameters within the river water was based on existing knowledge from previous literature; field study or experimentation to ascertain such fate and/or primary data of solute behavior are out of the scope of this study. Life cycle analyses of these water quality parameters in the river depends on aspects such as river water velocity, reaction dynamics, natural decay, possibility of interaction with the water from merging tributaries, etc., which could be addressed in future studies.

## 3. Results and Discussion

### 3.1. Present Scenario of Groundwater Quality with Respect to Arsenic Pollution in Ganges River Basin

#### 3.1.1. Occurrence of Arsenic in Groundwater

**Figure 2:**
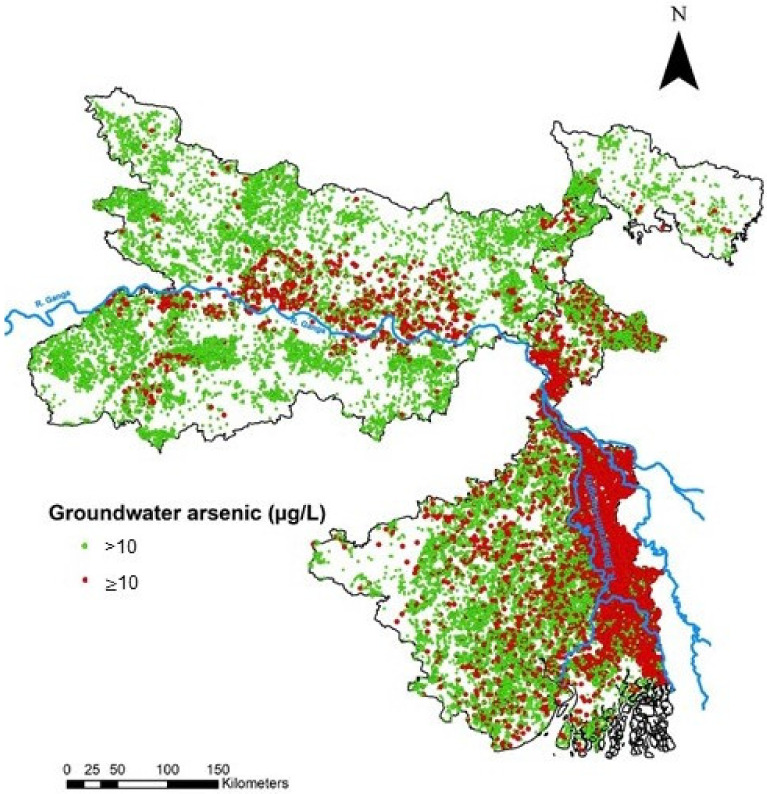
Groundwater arsenic concentration from field-observed groundwater data for the states of Bihar (*n* = 112,308) and West Bengal (*n* = 405,910).

Elevated levels of As in groundwater were reported first in 1976 from the state of Haryana [31] but came under attention when reported from West Bengal in 1983. Since then, elevated As has been reported in numerous tube wells in the country. Our compiled dataset of 428,951 field-observations across the study area shows that vast areas of the states of Bihar and West Bengal, lying in the central and lower Gangetic basin, respectively, have high groundwater As concentrations (≥10 µg/L) and interestingly these areas follow the Himalayan mountain front, with persistent existence in the Himalayan piedmont of the foothill aquifers of these states. About 26% of the areal extent of West Bengal and 21% of Bihar were found to be contaminated with elevated groundwater As. Some areas from the Bhagalpur and Darbhanga districts of Bihar and Maldah, Nadia and South Parganas districts of West Bengal are found to have groundwater As concentrations above 100 µg/L, which is well above the permissible limit of 10 µg/L. The most affected areas with groundwater elevated As were located in the central and eastern parts of Bihar and the east, central and south-eastern parts of West Bengal, whereas the areas with no risk of groundwater As pollution were observed in the west, north and southern parts of Bihar and the north and south-western parts of West Bengal. Besides such regional distribution of As in groundwater in the study area, local-scale variation exists. In the south-eastern parts of West Bengal, which lies in the delta of the Ganga river, local-scale variations such as higher occurrence of high groundwater As in the east bank than in the west bank of the Bhagirathi-Hooghly river have also been reported [14]. Our compiled dataset of field-observed groundwater As also shows similar variations in this region (Figure 2).

#### 3.1.2. Prediction of Groundwater Arsenic Concentration Using Machine Learning Based Approach

**Figure 3:**
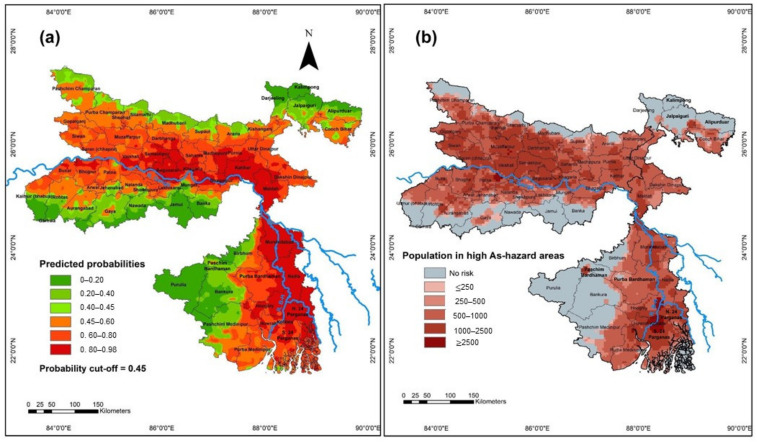
Map showing (**a**) Random forest predicted probabilities of groundwater arsenic (As) exceeding the permissible limit of 10 µg/L and indicating the probability cut-off of 0.45. (**b**) Population in predicted high risk areas of groundwater arsenic.

The probability cut-off of 0.45 was determined to classify the high and low risk probabilities of groundwater arsenic to be ≥10µg/L (i.e., probabilities ≤0.45 were low risk and >0.45 were high risk) (Figure S1). The RF model predicted significantly higher extents of areas possibly affected with elevated groundwater As and, consequently, predicted fewer areas to be at lower risk. The model predictions indicate that about 70% and 69% of the areal extent of Bihar and West Bengal, respectively, have high probability of being affected with groundwater As contamination, indicating a significantly lesser areal extent without risk of elevated As concentrations in groundwater (Figure 3). The areas predicted to be with a high probability of having groundwater As above 10 µg/L by the RF model are the central and eastern parts of Bihar and the south-eastern and south-western parts of West Bengal. Areas predicted to be at no risk are north and southern Bihar and north and south-western West Bengal. These predictions match field-observed data of As occurrence in groundwater (further model performance results can be found in Mukherjee et al., 2020) [24]. Districts such as Gaya, Jamui and Nawada in Bihar, and Alipurduar, Bankura, Darjeeling, Jalpaiguri, Paschim Bardhaman and Purulia are predicted to have very little or no risk of groundwater arsenic contamination (Figure 3). The relative variable importance shown by the RF model indicates that geotectonic, percentage of groundwater-fed irrigated area, elevation, sand content in the topsoil and groundwater depth are the most important predictors of groundwater As. These variables are indicators of the various (hydro)geological, (bio)geochemical, and anthropogenic processes that govern the distribution of As in groundwater. The geotectonic reflects influence of geodynamic processes on As especially in the Ganga river basin encircled by the Himalayan orogenic system [8,10] and possibly explain the distribution of high groundwater As following the Himalayan mountain front. Another important variable is that the percentage of the groundwater-fed irrigated area is a proxy for heavy abstraction of groundwater by pumping for irrigation in the majority of areas in these two states. Higher groundwater abstraction significantly increases hydraulic gradients and consequently induces higher As levels flushing into the aquifer. This increases the risk of encountering elevated As levels in groundwater. Together these factors and possibly several others are the controlling mechanism for high groundwater As in vast areal extents of the states of Bihar and West Bengal and, consequently, threaten the availability of safe water resources for the large population residing in these areas, where people are largely dependent on groundwater to meet their agricultural and drinking water requirements.

#### 3.1.3. Population Exposed to Elevated Groundwater Arsenic Concentration

Estimation of population exposed to elevated groundwater As was performed using the compiled dataset and the model predictions of groundwater As distribution at a resolution of 1000 m × 1000 m when considering the 2015 projected population. The population with no risk of high As levels from the compiled dataset of field-observations was found to be 80% and 72% of the total population for Bihar and West Bengal, respectively, i.e., approx. 83 million and 63 million respectively, while the population exposed to elevated groundwater As was ~21 million for Bihar and ~28 million for West Bengal, i.e., about 20% and 31% of the total population of the states, respectively (Figure 3). However, the model predictions of As distribution indicates much higher risk. The estimation from the model predicted distribution of elevated As in groundwater shows ~72 million for Bihar and ~70 million for West Bengal, i.e., about 69% and 76% of the total population of the states respectively may possibly be at risk due to elevated groundwater As. These estimations provide an understanding of the distribution of As in groundwater and the associated risk of As contamination within the study area.

### 3.2. Observations and Evaluation of Ganges River Water Quality

#### Impact of Lockdown in Arsenic Affected Parts of Ganges River Basin

**Figure 4:**
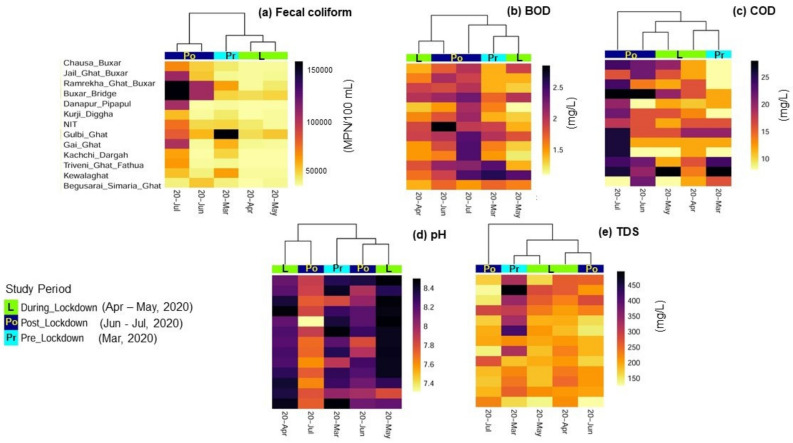
Heatmap of Pearson correlation based on average abundances of (**a**) Fecal content (FC) (MPN/100 mL), (**b**) Biochemical oxygen demand (BOD, mg/L), (**c**) Chemical oxygen demand (COD, mg/L), (**d**) pH and (**e**) total dissolved solids (TDS, mg/L) for the middle reaches of the Ganges river from March to July 2020.

Fecal coliform concentration in the middle reach of the Ganges river showed a distinct difference between the three different study periods (Table 1). During the lockdown, i.e., in the month of April and May 2020, fecal coliform (FC) concentration decreased compared to the pre- and post- lockdown period in thirteen locations of the middle reach of the Ganges. The average fecal coliform concentration was 100,000 MPN/100 mL (Figure 4, Table S2) during the pre-lockdown period, which decreased to <10,000 MPN/100 mL during the lockdown. The decrease in FC during the lockdown may be linked to the smaller built-up area and more croplands in the middle reach of the Ganges (Figure 1). Higher built-up areas may be linked directly to proper municipal sanitation systems which lead to an increase in sewage effluents in the Ganges, further increasing FC in the river. The lockdown period caused the stoppage of many daily urban activities, thus reducing sewage generation. This reduction of municipal sewage generation during lockdown may be directly linked to a decrease in FC during the lockdown. However, the post-lockdown period FC increased by 80% (±1.2) on average (150,000 MPN/100 mL on average) compared to the lockdown period. This increase of fecal coliform concentration in the post-lockdown period may indicate the increase of municipal sewage and the reopening of offices. Public health interventions primarily aim to prevent the population travelling from an affected area to other areas. There have been several studies on migration and its impact on the spread of this pandemic. It was observed that after the announcement of the country-wide lockdown due to COVID-19 in India at the end of March 2020, there was a sudden gathering of migrant workers from across the city of Delhi, Uttar Pradesh, Bihar and other states. It has been observed that the return of migrant workers was initiated at the end of May. Over two lakhs migrant workers returned to Bihar in the post-lockdown period [32,33]. This increase in population due to the return of migrant workers to Bihar may also have increased FC. Apart from FC, COD showed a distinct cluster among three different study periods (Figure 4). COD was found to be higher in the post-lockdown period compared to the lockdown period. In urban areas near Patna, Bihar, COD increased by 13% (22 mg/L ± 0.3) compared to rural/agricultural areas (19 mg/L ± 0.78). This increase of COD in the post-lockdown period may have been caused by the reopening of industries and factories. Unlike COD, biochemical oxygen demand (BOD), TDS, and pH did not show distinct clusters between the three study periods. However, BOD increased by 21% (±1.21) on average in the post-lockdown era compared to the lockdown period. A decrease in municipal sewage during lockdown increases the dissolved oxygen (>5 mg/L, WBPCB 2020), which may have led to a decline in BOD during the lockdown period, which supports our previous hypothesis. An earlier study by Dutta et al. (2018) also suggests that this increasing value of dissolved oxygen may also be attributed to a decrease of waste discharge from different non-point sources [34]. Though there is no distinct cluster between the three different study periods for Ganges river water pH, it has been observed that during the lockdown the pH of the middle reach of the Ganges river was alkaline (8.4 on average). However, after lockdown, specifically in July 2020, the pH decreased to 7.3 (Figure 4). This decrease in pH further supports the fact that the reopening of industries in the post-lockdown period led to an increase in industrial effluent discharge into the Ganges river, which eventually decreased the river water pH [35].

**Figure 5:**
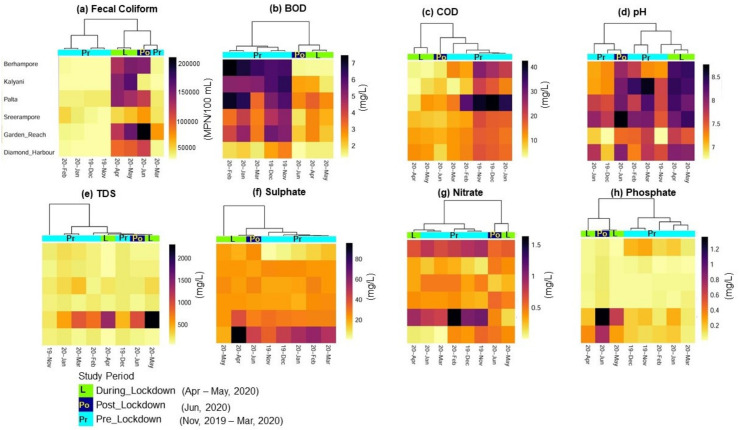
Heatmap of Pearson correlation based on average abundances of (**a**) FC (MPN/100 mL), (**b**) BOD (mg/L), (**c**) COD (mg/L), (**d**) pH, (**e**) TDS (mg/L), (**f**) sulfate (mg/L), (**g**) nitrate (mg/L) and (**h**) phosphate (mg/L) across the lower reaches of Ganges river from March to July 2020.

An increase of fecal coliform concentration was observed during the lockdown period compared to the pre-lockdown era in the lower reach of the Ganges (Table 2). There is a distinct difference between clusters among the three different study periods. During the lockdown period, FC increased by 94.2% (± 0.14) on average (150,000 MPN/100 mL on average) compared to the pre-lockdown period (10,000 MPN/100 mL on average). However, in the post-lockdown period, there is only a 3.5% (±0.25) decrease in FC (Figure 5). This result is opposite to that observed in the middle reach of the Ganges. This difference in results may be associated with differences in land-use patterns between the lower and middle reaches of the Ganges (Figure 1). The higher built-up area in the lower reaches of the Ganges uniquely influenced changes during the lockdown period. The higher urban and industrial settings in the lower reach increased both domestic sewage and industrial effluents in the river. The increase in FC during lockdown in the lower reach may be related to the decrease in antagonistic compounds released from industries or riverine transports which are more dominant in the inter-tidal reaches of the Ganges. The post-lockdown decrease in FC can be linked to the initiation of effluent disposal from the re-opened industries and riverine transport system. This hypothesis can be further strengthened by studying a particular location (Garden Reach station) in the lower reach. The FC concentration near Garden Reach station, located in the metropolitan city of Kolkata, showed the highest increase of FC during the post-lockdown period. This can be related to the discharge of effluents from about fifteen drains in the upstream of this station. These drains mostly contain domestic sewage [34]. However, in the pre-lockdown period, industrial effluent drains were also open along with domestic sewage effluents. The presence of industry effluents in the Ganges river showed a negative impact on fecal coliform growth [36,37]. BOD and COD showed clear clusters between the three study periods. Interestingly, both BOD and COD decreased by 58.1% (±0.03) and 55.7 (±0.04)%, respectively, during the lockdown period. The post-lockdown period did not show any significant differences (Figure 5). The decrease of industrial effluent in the lower reach of the Ganges river increased the dissolved oxygen level resulting in low BOD and COD during the lockdown period. Phosphate, TDS, and pH did not show any significant differences. However, sulfate showed distinct clustering between the three study periods. Due to enhanced domestic sewage, the highest concentration of sulfate was observed in the Garden Reach station, which may be further corroborated with FC. An average 14% (±1.02) increase in sulfate concentration was observed during the lockdown period in the lower reach of Ganges (Figure 5). Further, extreme climatic events like the cyclone “Amphan” between 19–20 May 2020 resulted in catastrophic impacts in southern West Bengal, resulting in possible sea water intrusion in this area. It is expected that the Garden Reach and Diamond Harbour stations, which are within the Gangetic estuary of the Bay of Bengal (BoB), would experience a tidal stormwater surge from BoB during such extreme events, and hence possibly reflected in the evolved river water chemistry, as observed during the lockdown. Elevated concentration of sulfate in seawater may be one of the possible sources for the observed higher sulfate concentration in the lower reaches of Ganges river water during lockdown period. The decrease of industrial activities may have increased dissolved oxygen in river water. However, due to lack of treated effluent, only municipal waste contributed sulfate during the lockdown period. Nitrate concentration was also not significant between the three different study periods.

### 3.3. Impact of Natural and Human Factors on Lockdown River Water Quality 

#### 3.3.1. Rainfall and River Flow

**Figure 6:**
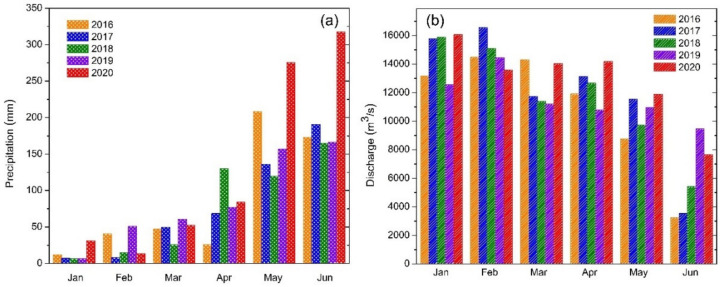
(**a**) Monthly mean rainfall data for the lower reaches of the Ganges river during 2016 and 2020. (**b**) Monthly mean Ganges river discharge from satellite-based estimates at a lower reach location (24.974°N, 87.975°E).

Rainfall rates show their highest monthly averages in January, May, and June during 2020; on the other hand, rainfall rates are found to be near normal or even lower than normal in February, March, and April in 2020 (Figure 6a). On comparing the rainfall patterns in January and June during the last few years, a consistent trend has not been observed in the lower reaches (Figure 6a). However, comparatively lower rainfall rates in March and April of 2020 do not support the dilution effect. The rainfall rates, therefore, do not explain the sudden change in water quality parameters during March and April of 2020.

Figure 6b depicts the increased discharge of waterflow in the Ganges river during lockdown due to less industrial consumption. In comparison to the previous year, discharge increased by approximately 15% during the lockdown period. The enhanced discharge may be construed to have been obtained from increased groundwater baseflow discharge, which typically is reduced due to enhanced irrigation abstraction [14]. The baseflow might have increased due to reduction of agricultural irrigation groundwater withdrawal. Low BOD and COD levels in the middle and lower reaches of the Ganges river, indicates the decreased industrial activities, and release of excess river water from reservoirs also increases the dissolved oxygen in river water. However, the elevated municipal sewage and hospital waste during lockdown clearly showed a higher fecal coliform concentration in the lower reaches. According to the Central Water Commission, Ministry of Jal Shakti, Govt. of India, it was observed that the storage of reservoirs in the Eastern region for the month of May 2020 was 40% higher than the ten-year average storage [38,39,40] (A possible reason for increased storage during lockdown might be reduced agricultural and irrigation activities during lockdown.

#### 3.3.2. Land Use and Land Cover

Land use and land cover play an important role in influencing the pollution levels in the river. In the lower reaches of the Ganges, where the urban setting is much more prominent and widespread, proper drainage and municipal sewage structure often lead to an increase in waste disposal in the river. In addition to this, widespread urban settings are also accompanied by a diverse industrial system, which often drains its waste into the river. This makes the river pollution level much higher in urban and peri-urban settings compared to rural settings. In the middle reaches of the Ganges, where, comparatively, urban settings are less prevalent, the chances of chemical and fecal pollution are lower. Rural systems lack proper drainage and sewage systems, and drain waste directly into the river. Rural households are mostly equipped with pit latrines rather than proper channelized sanitary pipelines. The presence of pit latrines often deters transport of fecal waste to the river, thus lessening fecal pollution. Though, eventually, fecal waste from pit latrines can leach to groundwater, which may consequently reach the river, the fate and transport of fecal waste through groundwater remains an open question. This holds true for other chemical waste, which may be generated from agricultural fields in the rural setting. Though studies show leaching of pesticide and other poly-aromatic hydrocarbons in the groundwater from agricultural fields [39], the time needed for these hazardous compounds to reach the river is not well understood.

**Figure 7:**
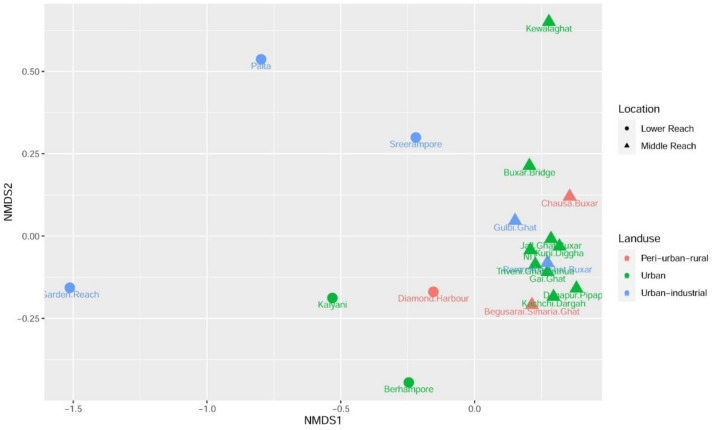
Non-metric multidimensional scaling (NMDS) of the Euclidean distance of the four measured parameters (pH, BOD, COD, and fecal coliform) during lockdown across all the locations from the lower and middle reaches of the Ganges river.

NMDS analysis based on the Euclidean distance matrix also yielded similar results (Figure 7). During the lockdown, clear partitioning of the locations from the middle reach and the lower reach of the Ganges was observed. It was interesting to note that locations from the lower reach were more scattered compared to locations from the middle reach. This was more prominent for locations from urban-industrial settings (Garden Reach, Palta, and Sreerampore) of the lower reach. The scattered positioning of these locations signifies distinct patterns of the measured parameters (pH, BOD, COD, and fecal coliform) across these regions. This can be supported by the presence of different industries in these three regions. In the lower reach, the locations from the urban settings (Kalyani and Berhampore) displayed close grouping, indicating similar patterns. In the middle reach of the Ganges, 12 out of 13 locations are tightly clustered, showing much less variability in the middle reach compared to the lower reach. The higher variability of the measured parameters in the lower reach compared to the middle reach may be attributed to the intertidal zone, higher motorized riverine transport, and the diverse industrial setting in the lower reach.

#### 3.3.3. Human Influence in Changing River Water Quality

Previous literature has demonstrated that anthropogenic activities are considered as one of the key drivers of surface water pollution [41,42]. The Ganges river is widely known to be one of the most polluted rivers in the world, with a large pollution load being introduced from various industrial effluents, settlement sewerage discharge and agricultural outfall. According to the national status of wastewater generation & treatment (ENVIS Centre on Hygiene, Sanitation, Sewage Treatment Systems and Technology, Govt. of India), estimated sewage generated as of 2016 in Bihar and West Bengal were 1879 and 4667 Million Liters per Day (MLD), respectively [43]. As of 2019, the estimated sewage generation in Bihar and West Bengal were 1270 and 3142 MLD, respectively [39]. According to Dutta et al. (2020), during the lockdown period in April and May 2020, the industrial pollution and several non-point source pollution levels were reduced [44].

During the lockdown period, the primary industrial sources of pollution that may affect river water quality, such as industrial wastewater discharge, crude oil, and heavy metals deposition, had dwindled or completely stopped [42]. However, discharge of plastics, municipal waste such as household/domestic sewage, and hospital wastes increased manifold. Our recent studies have shown the presence of several persistent organic pollutants (PoPs) in unacceptable concentrations. More than ten insecticides and five polycyclic aromatic hydrocarbons (PAHs), which are typically associated with industrial waste or urban combustion, were detected in the lower reaches of Ganges river [45]. Among the detected PoPs in Ganges river water, some of the predominant PAHs were naphthalene, fluoranthene and phenanthrene along with insecticides such as malathion, lindane, etc. [46]. Due to the unavailability of continuous data, quantitative analyses were not possible; however, PoPs concentration data, collected from the West Bengal Central Pollution Control Board (WBPCB) for the lockdown period, suggest that pesticides and PAHs concentration are below detection limit or have non-traceable concentrations in the studied lower reach of the river.

To understand the influence of lockdown on FC for the lower reach of the Ganges river, we have regressed the data with fixed effect panel data models. A significant impact of both BOD and COD on FC was observed (the coefficient associated with BOD, β_BOD_ = −0.02 with t-statistics—3.5, *p* < 0.01; and COD, β_COD_ = −0.003 with t-statistics—2.02, *p* < 0.01), suggesting that FC improves with the decrease of BOD and COD in the lower reach of the Ganges during lockdown phases. Effect of pH on FC was not significant during lockdown phases (β_pH_ = −0.05 with t-statistics −1.35, not significant) (Table 3). However, sulfate showed significant negative impact on FC (β_SO4_ = −0.03 with t-statistics −2.32, *p* < 0.01) in the lower reach of the Ganges river except for the Garden Reach station (β_SO4_ = 0.05 with t-statistics –3.41, *p* < 0.01) during the lockdown phase (Table 3). Panel data analysis indicates BOD is highly influenced FC during the lockdown. As BOD is the amount of oxygen consumed by bacteria in the decomposition of organic material, this indicates the higher availability of dissolved oxygen in the river stream during the lockdown. Reduction of industrial activities and riverine transport decreases the organic effluent discharge in the river stream, which enhances the substrate availability for fecal coliform growth. Apart from these water quality parameters, exogenous parameters (β_SO4_ = −4.018 with t-statistics –20.32, *p* < 0.01) such as effluent discharge, pesticide concentration and riverine transport also influence the fecal coliform growth in the lower reach of the Ganges river during lockdown.

### 3.4. Groundwater-Sourced Drinking Water/Ganges River Sourced Drinking Water

Since the mid-1980s, several policies have been implemented to restore the Ganges river; however, none of them have provided the desired outcome. Prominent programs included the GAP and NGM that made consolidated and integrated efforts towards river conservation [14]. However, with evolving knowledge it is becoming more and more obvious that a concerted effort to revive the river should include scientifically prudent, human-centric policies that would restore a regional ethos and cater to local needs.

The large groundwater arsenic dataset used in this study provides a novel method of determining and predicting polluted regions in the study area. Simultaneously, the studied parameters of river water quality, including fecal coliform, BOD, COD, pH, sulfate, phosphate, nitrate, TDS and some of the Persistent Organic Pollutants, provide indicators of water quality modification over time for lockdown and non-lockdown periods. Thus, the observed measurements and their analyses provides knowledge to deduce the impacts of potential future amendments to human practices in improving the river-sourced drinking water sources in these known groundwater arsenic-polluted areas.

Thus, a future roadmap to rejuvenate the river may be envisaged from the observation of water quality changes from the lockdown period, with adaptation of insights and integration of diligence, long-term maintenance and sustainability of solutions, along with social integration of technology using community participatory approaches:

1. Efficient disposal of hazardous industrial and urban effluents like oils, anti-freeze, paint, solvents, cleaners, preservatives, and biomedical, which should not be directly introduced to the river water.
2. Introduction of zero-discharge waste-water treatment facilities with integration of existing traditional water remediation techniques with emerging, locally adaptive technologies such as phytoremediation, bioremediation, riverbank filtration, etc.
3. Reduce or eliminate use of fertilizers and chemical herbicides and pesticides as these chemicals are the dominant source for PoPs, as well as elevated nitrogen and phosphorus pollution and toxic runoff [43,44].
4. Enhanced sanitation and structured and planned septic systems need to be in place and measures need to be taken to restrict defecation outflow to the river water.
5. Irrigational water preservation through regulated groundwater usage may be adapted through cropping of native food crops and reduction of water-guzzling cash-crops.
6. Enhance natural groundwater infiltration and recharge through resurface with permeable land cover in urban brawls.
7. Knowledge dissemination through participatory local training and utilization of ground knowledge for long-term sustainable rejuvenation.
8. Introduction and implementation of a stringent regulatory framework for river water quantity and quality, replenishment and rejuvenation.

Hence, in order to rejuvenate the river, the groundwater system interacting with and sustaining the river flow needs to be revived. While this may be engineered through replenishment of aquifers following detailed hydrogeological investigation, conjunctive surface and groundwater policy also needs to be framed and implemented. This would need a huge public investment and incentives, which would not only cater for Sustainable Development Goal 6 (SDG 6), but could also lead to poverty alleviation (SDG 1), provide food through sustainable use of the river water (SDG 2) and promote health for the residents of the Ganges river basin (SDG 3).

## 4. Conclusions

The ravaging of the global socio-cultural and economic framework from the beginning of 2020 due to the Covid-19 pandemic resulted in a longterm setback to plans for attaining sustainable development. Across the globe, countries, including India, imposed stringent mass quarantine and lockdown to curb the transmission of the pathogen. India, one of the most affected countries, experienced an unprecedented 68-day lockdown between end-March and May 2020. The lockdown forced suspension of normal life, including industrial activities, transportation, etc. While the efficacy of such a lockdown is debatable, it provided an inadvertent benefit in positively impacting the environment.

In the present work, the impact of the lockdown on sustainable drinking water availability in the Ganges river basin, widely known for groundwater arsenic pollution as well as the severely deteriorated Ganges river water, is assessed. Groundwater arsenic measurements from a large number of drinking water wells across the study area and time-variant river water qualities including fecal coliform, BOD, COD, pH, sulfate, phosphate, nitrate, TDS and some Persistent Organic Pollutants were used to determine region-specific drinking water availability in areas with known arsenic pollution at normal (non-lockdown) and paused human activity (lock-down) periods.

The present study, along ~700 km stretches of the middle and lower reaches of the Ganges river basin in the Indian states of Bihar and West Bengal, evaluated drinking water wells for groundwater arsenic concentrations, as well as water quality data from 19 stations, collected monthly from January to July 2020, to understand the availability of sustainable drinking water sources. Thus, the data provides a unique opportunity to examine any influence of suspension of normal life and industrial activities on drinking water quality, in contrast to pre- and post-lockdown periods.

The compiled dataset of field-observed groundwater As revealed that the west, north and southern parts of Bihar and the north and south-western parts of West Bengal were not affected with groundwater As pollution, with approximately 83 million (80% of the total population) and 63 million (63% of the total population) of Bihar and West Bengal, respectively, with no risk of groundwater As contamination exposure. The RF model predictions indicated a lesser areal extent to be possibly at low/no risk, located in the north and southern parts of Bihar and the north and south-western parts of West Bengal, with about 31% and 21% of the total population possibly at no risk of As contamination exposure for Bihar and West Bengal, respectively. Further, the lockdown period demonstrated that there has been a general improvement in water quality in both the middle and lower reaches of the the Ganges river, potentially due to less discharge of industrial effluent and agricultural outflow. While, due to the lack of a major industrial hub and the dominance of rural agrarian communities, agriculture-borne pollutants is more prevalent in the middle reaches, urban waste and industrial-borne pollution dominate in the lower reaches. Thus, the other source-sensitive water quality parameters are also affected by such influencing factors. The concentrations of BOD and COD decreased across the river. During lockdown, the pH of the Ganges increased across different locations, which may be related to the interruption of industrial activities. However, interestingly, TDS did not document much change in the middle reach and somewhat decreased in the lower reach, potentially suggesting the natural weathering-sourced major cations dominating the hydrochemistry.

These observations of the availability of groundwater arsenic-safe drinking water wells and inadvertent water quality changes during lockdown thus provided a near-hypothetical scenario for evaluating water quality in a time-window like that of the pre-industrial era. This situation provides a roadmap by which, the ground-reality geospatial distribution and variance in hydro-chemical fate of the observation of pollutants may be integrated with scientifically prudent, adaptive river management strategies and spatially targeted remediation, which should lead to an understanding of safe and sustainable drinking water availability in the polluted river basin through innovative social engineering and technological interventions. Thus, there is a cautious optimism#; however, it is evident that the preservation of the water quality of both groundwater and the river is mostly dependent on the policy makers, business stakeholders, and common residents dwelling on the banks of the river.

## Figures and Tables

**Table 1:** Average concentration/value of different parameters at thirteen locations during lockdown period in middle reaches of Ganges river.

Parameter	During Lockdown (Average)
Faecal Coliform Most Probable Number (MPN/100 mL)	5000
Biochemical oxygen demand (BOD)	1.6
Chemical oxygen demand (COD) (mg/L)	14.34
pH	8.34
Total dissolved solids (TDS) (mg/L)	212.4
Sulphate (mg/L)	No Data
Phosphate (mg/L)	No Data
Nitrate (mg/L)	No Data

MPN stands for Most Probable Number.

**Table 2:** Average concentration/value of different parameters at six locations during lockdown period in lower reaches of Ganges river.

Parameter	During Lockdown (Average)
Faecal Coliform (MPON/100 mL)	180,000
BOD	2.2
COD (mg/L)	8.25
pH	8.14
TDS (mg/L)	302.2
Sulphate (mg/L)	29.61
Phosphate (mg/L)	0.15
Nitrate (mg/L)	0.42

**Table 3:** Panel data analysis showing fixed effect of BOD, COD, pH and sulphate on FC during lockdown period (Apr–May 2020).

Dependent Variable: FC **	
Independent Variable	Fixed Effect
BOD	−0.02
	(−3.5) *
COD	−0.003
	(−2.02) *
pH	−0.05
	(−1.35)
Sulphate	−0.03
	−2.32
Sulphate in Garden Reach	0.05
	3.41
Constant	−4.018
	(−20.32) *
No. observation	24
r^2^	0.84

* t-statistics values are shown in parenthesis. ** FC: Fecal coliform concentration.

## Data Availability

Data available on request.

## Supplementary Materials

The following are available online at https://www.mdpi.com/1660-4601/18/6/2832/s1, Table S1: Sampling locations showing three different land use patterns across middle and lower reaches of Ganges river., Table S2: Summary of observations for the changes of water quality parameters in comparison of lockdown months (April–May 2020) to pre-and-post lockdown periods of 2020. Figure S1: (a) Binary risk map showing areas with high and low risk of groundwater As prepared using the RF predicted probabilities and cut-off 0.45. (b) RF model sensitivity and specificity vs. cut-off (adopted from Mukherjee et al., 2020).
