# Supplementary material for: Impact of Covid-19 Lockdown on Availability of Drinking Water in the Arsenic-Affected Ganges River Basin
Source: Int J Environ Res Public Health. 2021 Mar 10;18(6):2832. doi: 10.3390/ijerph18062832 (PMC8001881; doi:10.3390/ijerph18062832)
Supplement: Supplementary file 1 [file ijerph-18-02832-s001.pdf]

International Journal of
Environmental Research and Public Health

MDPI

Supplementary Information

Table S1. Sampling locations showing three different land use patterns across middle and lower reaches of Ganges river.

	Places	Land use
Middle Reach	Gai Ghat	Urban
	Gulbi Ghat	Urban-industrial
	Jail Ghat Buxar	Urban
	Kachchi Dargah	Urban
	Kurji Diggha	Urban
	NIT	Urban
	Ramrekha Ghat Buxar	Urban-industrial
	Kewalaghat	Urban
	Triveni Ghat Fathua	Urban
	Buxar Bridge	Urban
	Chausa Buxar	Peri-urban-rural
	Begusarai Simaria Ghat	Peri-urban-rural
	Danapur Pipapul	Urban
Lower Reach	Garden Reach	Urban-industrial
	Kalyani	Urban
	Palta	Urban-industrial
	Sreerampore	Urban-industrial
	Berhampore	Urban
	Diamond Harbour	Peri-urban-rural

Table S2. Summary of observations for the changes of water quality parameters in comparison of lockdown months (April-May 2020) to pre-and-post lockdown periods of 2020.

Parameters	Middle Reach	Lower Reach
Faecal Coliform	Decreased - (28.6±1.2%)	Decreased - (3.5 ±0.25%)
BOD	Decreased - (32 (± 1.01%)	Decreased - (58.1 ± 0.03%)
COD	Decreased - (9.2 ±0.3%)	Decreased – (55.7 ± 0.04%)
pH		Increased
TDS	Similar	Decreased
Sulphate	No data	Increased + (14 ±1.02%)
Phosphate	No data	Increased (2.03 ±0.3%)
Nitrate	No data	Similar

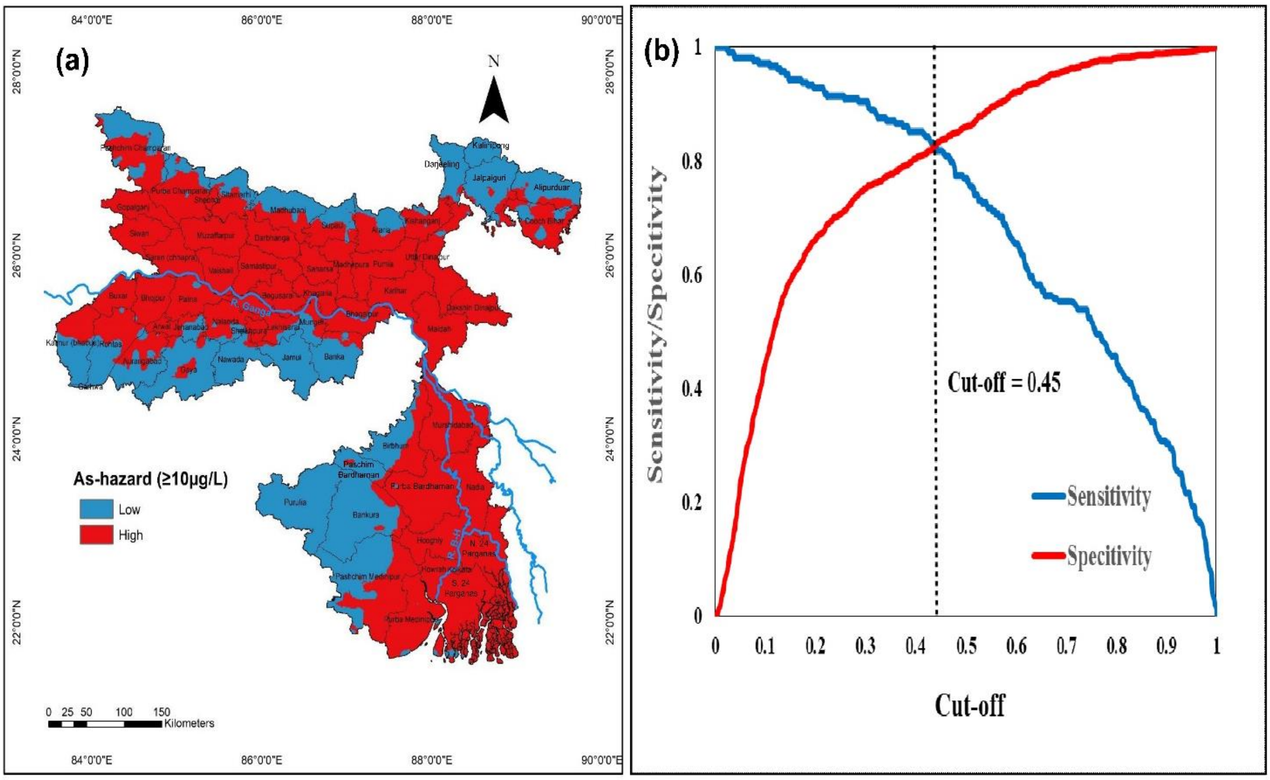

Figure S1. (**a**) Binary risk map showing areas with high and low risk of groundwater As prepared using the RF predicted probabilities and cut-off 0.45. (**b**) RF model sensitivity and specificity vs. cut-off (adopted from Mukherjee et al., 2020).
